# Bactericidal Property of Oregano Oil Against Multidrug-Resistant Clinical Isolates

**DOI:** 10.3389/fmicb.2018.02329

**Published:** 2018-10-05

**Authors:** Min Lu, Tianhong Dai, Clinton K. Murray, Mei X. Wu

**Affiliations:** ^1^Wellman Center for Photomedicine, Massachusetts General Hospital, Harvard Medical School, Boston, MA, United States; ^2^First Area Medical Laboratory, JBSA-Fort Sam Houston, Houston, TX, United States

**Keywords:** oregano oil, *Pseudomonas aeruginosa*, *Acinetobacter baumannii*, MRSA, biofilms, burn wound, mouse model, bioluminescence imaging

## Abstract

Development of non-antibiotic alternatives to treat infections caused by multidrug-resistant (MDR) microbes represents one of the top priorities in healthcare and community settings, especially in the care of combat trauma-associated wound infections. Here, we investigate efficacy of oregano oil against pathogenic bacteria including MDR isolates from the combat casualties *in vitro* and in a mouse burn model. Oregano oil showed a significant anti-bacterial activity against 11 MDR clinical isolates including four *Acinetobacter baumannii*, three *Pseudomonas aeruginosa*, and four methicillin-resistant *Staphylococcus aureus* (MRSA) obtained from combat casualties and two luminescent strains of PA01 and MRSA USA300, with a MIC ranging from 0.08 mg/ml to 0.64 mg/ml. Oregano oil also effectively eradicated biofilms formed by each of the 13 pathogens above at similar MICs. Transmission electron microscopy (TEM) and scanning electron microscopy (SEM) revealed that oregano oil damaged bacterial cells and altered the morphology of their biofilms. While efficiently inactivating bacteria, there was no evidence of resistance development after up to 20 consecutive passages of representative bacterial strains in the presence of sublethal doses of oregano oil. *In vivo* study using the third-degree burn wounds infected with PA01 or USA300 demonstrated that oregano oil, topically applied 24 h after bacterial inoculation, sufficiently reduced the bacterial load in the wounds by 3 log_10_ in 1 h, as measured by drastic reduction of bacterial bioluminescence. This bactericidal activity of oregano oil concurred with no significant side effect on the skin histologically or genotoxicity after three topical applications of oregano oil at 10 mg/ml for three consecutive days. The investigation suggests potentials of oregano oil as an alternative to antibiotics for the treatment of wound-associated infections regardless of antibiotic susceptibility.

## Introduction

Skin wound infection is a widespread problem in both civilian and military healthcare settings. Skin wounds are particularly prone to bacterial infections because the wounds provide an ideal medium for bacterial proliferation and a portal of entry into the bloodstream and are direct exposure to the “dirty” environment. The infections can be readily treated with a variety of antibiotics if the bacteria involved are susceptible. However, there are only extremely limited or no treatment options when antibiotic resistant strains are involved in the wound infections, which occurs at a worrying speed. If the wound infection cannot be eliminated in a timely fashion, the infection would alter cellular metabolisms and induce persistent inflammation systemically that can predispose the patients to various complications and life-threatening sepsis (Fitzwater et al., 2003; Wang et al., 2018). For instance, burn wound infection outbreaks caused by multidrug-resistant (MDR) organisms emerged as a serious problem early in the course of Iraq military operations despite that the United States military has provided rapid and highly effective care for wounded soldiers (Scott et al., 2007; Vento et al., 2013). As a matter of fact, skin infections caused by MDR bacteria are the most common cause of morbidity and mortality in patients infected with MDR microbes and represent almost 61% of deaths of this infected population (Gomez et al., 2009).

Extensive uses of broad spectrum antibiotics are the single most important factor in evolution of bacterial resistance (Hampton, 2013). A number of studies have shown that the most frequently identified MDR strains of bacteria in nosocomial infections and on the battlefield are Gram-negative bacteria *Acinetobacter baumannii* and *Pseudomonas aeruginosa*, and Gram-positive bacterium methicillin-resistant *Staphylococcus aureus* (MRSA) (Scott et al., 2007; Calhoun et al., 2008; Li et al., 2014; Levin-Reisman et al., 2017). In addition, bacterial biofilms formed by MDR bacteria are the major obstacles in treatment of burn wounds (Bloemsma et al., 2008; Jiang et al., 2017). Bacteria within biofilms can be as much as 1,000 times more resistant to antibiotics and are responsible for recurrent antibiotic-resistant infections elsewhere in the body upon dissemination from the site of the biofilm (Ceri et al., 1999; Caraher et al., 2007). Currently, the only effective treatments available to fight these infections are older drugs like colistin, which are highly toxic and detrimental to the overall health of the patients (Crane et al., 2009). There is a pressing need for the development of non-antibiotic approaches to combat MDR microbes.

Essential oils (EOs) are a mixture of volatile constituents produced by aromatic plant/herbs. There are about 3,000 well-recognized EOs, of which 300 are generally recognized as safe (GRAS) to humans by the United States Food and Drug Administration (U.S. FDA) and have broad applications in food preservation, additives, and favors, perfume, cosmetic industries, antiseptic oral solutions, toothpastes, cleaner, and air fresheners for centuries (Pandey et al., 2017; Sakkas and Papadopoulou, 2017). These natural products are of particular interest as “green” antimicrobial agents because of their low-cost, biocompatibility, potential antibiofilm properties, and friendly to eukaryote cells and environment (Burt, 2004; Nostro et al., 2007; Kavanaugh and Ribbeck, 2012). Among these safe EOs, oregano oil has been shown to have a variety of activities such as antioxidant (Yan et al., 2016), anti-inflammatory (Ocana-Fuentes et al., 2010; Shen et al., 2010), anti-fungal (Akgul and Kivanc, 1988; Soylu et al., 2007), and anti-allergic (Benito et al., 1996). Its antimicrobial effect has been demonstrated *in vitro* cell culture, food systems studies (Lopez-Reyes et al., 2010; Soylu et al., 2010; Munhuweyi et al., 2017), and *in vivo* systemic infections (Manohar et al., 2001; Preuss et al., 2005). In the present study, we investigate effectiveness of oregano oil in inactivation of MDR bacteria isolated from combat casualties *in vitro* and bioluminescent strains of *P. aeruginosa* (PA01) and MRSA (USA300) in mouse burn wounds. Our study showed that oregano oil effectively inactivated various pathogenic bacteria and their biofilms irrespective of their antibiotic susceptibility. The study is the first *in vivo* attempt on the use of oregano oil for the treatment of burn wounds infected with clinically important MDR bacteria.

## Materials and Methods

### Chemical Constituents of Oregano Oil

Oregano oil was purchased from Bulk Apothecary (Aurora, OH, United States) and used throughout the study. To define the constituents of oregano oil, gas chromatography/mass spectrometry (GC-MS) analyses were carried out using an Agilent 6980 GC coupled to an Agilent 5973N MS and a fused-silica capillary column (HP-5MS: 30 m × 0.25 mm i.d., film thickness 0.25 μm). The initial column temperature was set at 60°C for 10 min and then increased at 3°C/min till 220°C. The temperature was held at 220°C for 10 min and raised to 240°C by increments of 1°C/min; the injector port temperature was 250°C with the carrier gas of helium at a flow rate of 0.8 ml/min. Ionization voltage of MS in the EI-mode was 70 eV and ionization source temperature at 250°C with a mass range of 35–465 amu. The volatile components were identified by comparison of their retention indices relative to *n*-alkanes (C6-C28) and mass spectra with those of authentic compounds by means of NIST and Wiley databases and with the Adams library spectra.

### Bacterial Strains

The antibacterial activity of oregano oil was tested against a panel of MDR bacteria isolated from combat casualties, including seven Gram-negative strains *A. baumannii* (AF0004, AF0005, IQ0012, and IQ0013) and *P. aeruginosa* (AF0001, IQ0042, and IQ0046), and four Gram-positive MRSA (AF0003, IQ0064, IQ0103, and IQ0211). All the bacterial isolates were obtained from San Antonio Military Medical Center under a Materials Transfer Agreement and demonstrated MDR according to the microbiology tests performed at the United States Army Institute of Surgical Research (**Table 2**). Amongst the *A. baumannii* and *P. aeruginosa* tested, only strains of IQ0012, IQ0013, and IQ0042 were susceptible to imipenem; the rest of strains were resistant to all antibiotics tested. The five MRSA strains were resistant to amikacin, ampicillin, cefazolin, cefoxitin, and erythromycin, but susceptible to gentamicin, levofloxacin, nitrofurantoin, and tetracycline. In addition, luminescent strains of *P. aeruginosa* (PA01) and MRSA USA300 were used in the *in vivo* study, allowing real-time monitoring of infection in the mouse burn wounds *in vivo* via bioluminescence imaging (Dai et al., 2013; Zhang et al., 2014; Wang et al., 2016).

### Determinations of Minimum Inhibitory Concentration (MIC)

In order to determine a MIC, a broth microdilution assay was employed as previously described (Joshi et al., 2010; Gao et al., 2011). Stock solution of oregano oil was prepared at 40 mg/ml in DMSO and twofold dilutions (0.04–1.28 mg/ml) of the stock EO in brain heart infusion (BHI) medium were added into 96-well plates for bactericidal tests. In each well, 20 μL of the suspensions containing 10^8^ CFU/ml of bacteria was added to 180 μL of the above medium containing oregano oil at varying concentrations. Medium supplemented with a similar amount of DMSO only severed as controls. The microplates were incubated at 37°C for 24 h and examined for bacterial growth. The first well without turbidity was determined as a MIC value. All assays were performed in triplicate.

### Antibiofilm Activity

Bacteria were incubated in trypticase soy broth (TSB) with 0.1% glucose at 37°C for 18 h, after which the cultures were harvested by centrifugation and washed twice with PBS. Bacterial suspensions with an optical density of OD_600_ equal to 0.1 in TSB were added to 96-well plates at 100 μL/well, followed by incubation at 37°C under static condition for 24 h to form biofilms. Oregano oil was added at indicated concentrations and incubated with the biofilms for 1 h, after which biofilms were washed twice with PBS and bacterial viability was determined using an Alamar Blue assay. TSB without bacteria was used as a negative control. All experiments were performed in triplicate.

### Assessment of Possible Resistance Development to Oregano Oil

To study any potential development of resistance to oregano oil, three representative strains (*A. baumannii* AF0005, *P. aeruginosa* IQ0042, and MRSA IQ0064) were propagated for 20 generations in the presence of sublethal doses of oregano oil as previously described (Li et al., 2014). Briefly, 200-μL aliquots of the bacterial suspensions (10^7^ CFU/ml) were inoculated into 96-well plates and exposed to the sub-MIC (2/3 MIC) of oregano oil at 37°C for 1 h, and the resultant bacteria were labeled as the first generation and tested for a MIC as above. The second generation was obtained by exposing the first generation to its sub-MIC for 1 h and determined for its MIC again. The procedure was repeated for up to 20 times. Oregano-resistance was determined by any significant increases in the MIC of successive generations.

### Transmission Electron Microscopy (TEM)

To determine bactericidal mechanism of oregano oil, *A. baumannii* AF0005 and *P. aeruginosa* IQ0042 were investigated as the representative strains for oregano-induced ultrastructural damages using transmission electron microscopy (TEM). Bacterial suspensions were fixed in 2.5% glutaraldehyde plus 2% paraformaldehyde overnight at 4°C. Fixed cells were collected by centrifugation at 4,000 × *g* for 5 min and rinsed with 0.1 M sodium cacodylate buffer (pH 7.2) for three times. After the final wash, hot agar was added to each pellet and the cell pellets were post-fixed in 2% osmium tetroxide for 1 h, dehydrated with a graded ethanol series, embedded in fresh Epon, and then polymerized at 60°C for 48 h. Ultra-thin sections were cut on ultramicrotome and collected onto 200 mesh bare copper grids. Samples were stained for 30 min with uranyl acetate and lead citrate and examined with a CM-10 TEM (Philips, Eindhoven, Netherlands).

### Scanning Electron Microscopy (SEM)

To investigate the ultrastructural changes of bacterial biofilms caused by oregano oil, scanning electron microscopy (SEM) was performed using the representative strains of *P. aeruginosa* IQ0042 and MRSA IQ0064. Briefly, biofilms of IQ0042 and IQ0064 were grown for 24 h on sterilized squares of ACLAR 33C (Electron Microscopy Sciences, Hatfield, PA, United States), and treated for 1 h with oregano oil at 0.75 mg/ml or 0.3 mg/ml, respectively. Untreated and oregano-treated biofilms were fixed at 4°C for 24 h in 0.1 M sodium cacodylate buffer containing 2.5% glutaraldehyde, 0.15% alcian blue, and 0.15% safranin O. The fixed biofilms were washed with 0.1 M sodium cacodylate buffer, infiltrated with 2% osmium tetroxide for 2 h, and dehydrated to 100% ethanol. The biofilms were dried using a critical-point dryer (Tousimis Research Corporation, Rockville, MD, United States), mounted on specimen stubs, sputter-coated with 10 nm Cressington 208 platinum (Cressington Scientific Instruments, Watford, United Kingdom), and examined on a S4800 SEM (Hitachi Ltd., Tokyo, Japan). Micrographs were acquired under high vacuum using an accelerating voltage of 3.0 kV.

### Animal

Female BALB/c mice at 8 weeks of age and 17–19 g were purchased from Charles River Laboratories (Wilmington, MA, United States). All animal procedures were approved by the Institutional Animal Care and Used Committees (IACUC) of Massachusetts General Hospital (Protocol 2014N000009) and were in accordance with guidelines of the National Institutes of Health.

### Treatment of Burn Infection in Mice by Oregano Oil

Mice were anesthetized with an intraperitoneal injection of ketamine-xylazine cocktail and shaved on the lower dorsal skin. The burn was introduced by a brass block (1 cm^2^) heated to thermal equilibration with boiling water prior to application of its extremity onto the shaved skin for 5 s, which generated a third-degree burn wound. Sterile saline was intraperitoneally administered at 0.5 ml/mouse to support fluid balance during recovery. Aliquots of 50 μL bacterial suspensions containing 5 × 10^6^ CFU in PBS were inoculated onto the burn 30 min after the injury and remained in place while the mice recovered from anesthesia. Luminescent strains of *P. aeruginosa* PA01 and MRSA USA300 were used as the causative pathogens in the study. At 24 h after bacterial inoculation when the biofilms were formed in the wounds, oregano oil was diluted with grape seed oil, which is commonly used for EO dilution in aromatherapy, at a final concentration of 5 or 10 mg/ml, and topically applied to the infected wounds.

Bioluminescence emission of the bacteria in the wounds was recorded in real time by a Lumina *in vivo* image system (IVIS) (PerkinElmer, Waltham, MA, United States). Bioluminescence images were acquired (60 s exposure, medium binning) at different time points after infection. During imaging, mice were anesthetized in chambers containing 2.0% isoflurane inhalant mixed with oxygen via an IVIS manifold placed within the imaging chamber. Bioluminescence was quantified with the Living Image software (Xenogen).

For measurement of bacterial burden, the infected burn wounds were collected after sacrifice of the mice on day 7 of bacterial inoculation. The collected tissues were homogenized in 2 ml sterile PBS. The resultant homogenate was serially diluted and spotted onto BHI agar plate containing Skirrow’s supplement (10 μg/ml vancomycin, 5 μg/ml trimethoprim lactate, and 2500 IU/L polymyxin B). The plates were then incubated at 37°C for 24 h and bacterial colonies were enumerated in a treatment-blind fashion.

### Gram Stain

Gram stain was carried out as previously described, with some modifications, to corroborate formation of PA01 biofilms in the infected wounds (Christensen et al., 2013; Wang et al., 2016). Briefly, at 24 h after bacterial inoculation, the infected wounds were excised, fixed in 10% phosphate-buffered formalin for 2 days, and then embedded in paraffin. Tissue sections were cut at 5 μm, de-paraffined, and rehydrated, followed by staining with 0.8% crystal violet in 1% sodium bicarbonate for 1 min and then in gram’s iodine for 2 min. After decolorization with acetone/alcohol = 1:1 (v/v), the tissue sections were counterstained with 0.1% safranin O for 2 min, washed, air dried, and mounted with permount (Fisher Scientific, Waltham, MA, United States). Sections were visualized by Hamamatsu NanoZoomer 2.0 HT and the images were processed using NDP viewer software.

### Toxicity of Oregano Oil to Mouse Skin *in vivo*

To evaluate any possible toxicity of oregano oil to the skin *in vivo*, mice were shaved on the low dorsal skin 24 h prior to application of oregano oil. Oregano oil *at* 10 mg/ml was applied topically on the shaved area once a day for three consecutive days. Mice treated with PBS served as negative controls. The mice were sacrificed 24 h after the final oregano oil application, and the skin was cross-sectioned using 8 mm biopsy punch for standard histological examination. The tissue sections stained with hematoxylin and eosin (HE) were visualized by Hamamatsu Nanozommer 2.0 HT and the images were processed using NDP viewer software.

DNA damage in oregano oil-treated skin was assessed using the DeadEnd Fluorometric TUNEL system (Promega, Madison, WI, United States), in which damage DNA undergoes end labeling with fluorophore as per the manufacturer’s instruction. Briefly, after deparaffinization and rehydration, the tissue sections were incubated with Proteinase K for 10 min to permeabilize the cells, washed, and stained with the TUNEL reaction mixture for 1 h at 37°C in a humidifies chamber. The sections were counterstained with DAPI to mark cell nuclei. Fluorescence images were captured using a FluoView FV1000-MPE confocal microscopy (Olympus Corporation, Tokyo, Japan). For the positive control, tissue sections were pre-treated with 10 unit/ml of RQ1 RNase-free DNase I for 10 min to induce DNA fragmentation before the sections were assayed by the TUNEL staining kit.

### Statistical Analyses

Data are presented as means ± standard deviations (SDs). Statistical significance was assessed with two-tailed Student’s *t*-test between two groups or one-way ANOVA for multiple group comparison. *P*-values of < 0.05 were considered statistically significant. All statistical analyses were performed using GraphPad Prism 7.0 (GraphPad Software).

## Results

### GC/MS Analysis of Constituents in Oregano Oil

Chemical ingredients of commercial oregano oil were identified by GC/MS analysis (**Table 1**). The twenty constituents accounted for 97.45% of the total amount of ingredients in oregano oil. The phenols carvacrol (72.25%) and thymol (6.62%), as well as the monoterpene hydrocarbons *p*-cymene (5.21%), γ-terpinene (4.12%), and α-pinene (1.21%) were the predominant components of oregano oil.

**Table 1:** Chemical composition (%) of the oregano EO determined by GC-MS analyses.

No.	Constituents	RI^a^	RI^b^	Peak area (%)^c^
1	α-Thujene	926	924	0.21
2	α-Pinene	939	932	1.21
3	Camphene	953	946	0.13
4	1-Octen-3-ol	982	974	0.23
5	Myrcene	991	988	2.12
6	α-Phellandrene	1002	1002	0.62
7	α-Terpinene	1018	1014	0.10
8	*p-*Cymene	1026	1020	5.21
9	Limonene	1031	1024	0.56
10	γ-Terpinene	1060	1054	4.12
11	Terpinolene	1092	1086	0.10
12	Linalool	1100	1095	1.21
13	Borneol	1165	1165	0.35
14	Terpinen-4-ol	1178	1174	0.56
15	Thymol	1295	1289	6.62
16	Carvacrol	1315	1298	72.25
17	Caryophyllene	1426	1417	0.66
18	α-Humulene	1456	1452	0.12
19	β-Bisabolene	1509	1505	0.35
20	Isocaryophyllene oxide	1585	1582	0.72
	Total			97.45


*^*a*^Retention index relative to C6-C28 *n*-alkanes on the HP-5MS capillary column. ^*b*^Retention index identification by using the Kovats index. ^*c*^Expressed as percentage of the total peak area of the chromatograms without correction.*

### Oregano Oil Effectively Inactivated Bacteria *in vitro* Irrespective of Antibiotic Sensitivity

**FIGURE 1:**
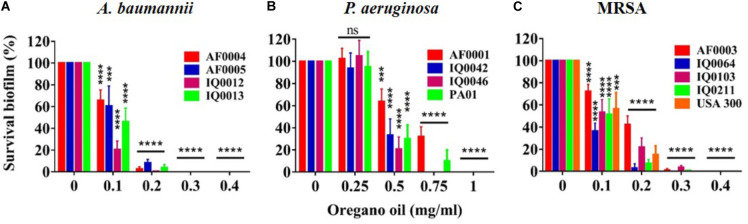
Bacterial viability of 24 h-old-biofilms formed by MDR strains of *A. baumannii* **(A)**, *P. aeruginosa* **(B)**, and MRSA **(C)** after 1 h treatment with oregano oil at indicated concentrations. The viability of bacterial biofilms was determined using an Alamar Blue assay. Data represent means ± SDs. ^∗∗∗^*p* < 0.001 and ^∗∗∗∗^*p* < 0.0001 in the presence vs. absence of oregano oil. ns, no significance.

The MIC values of oregano oil against the 13 bacterial strains are shown in **Table 2**. Oregano oil showed a significant antibacterial activity over PBS controls against *A. baumannii* strains of AF0004, AF0005, IQ0012, and IQ0013 and MRSA strains of AF0003 and IQ0211, with the MICs ranging from 0.08 to 0.16 mg/ml. The MICs were significantly lower than those MICs ranging from 0.32 to 0.64 mg/ml against strains of *P. aeruginosa* and MRSA strains IQ0064, IQ0103, and USA300 (**Table 1**). Oregano oil also exhibited similar antibacterial activities against established biofilms (24-h-old) formed by the 13 bacterial strains within 1 h, with complete inactivation of the biofilms of *A. baumannii*, *P. aeruginosa*, and MRSA at the concentrations of 0.3, 1.0, and 0.4 mg/ml, respectively, in good agreement with the MIC values for planktonic bacterial cells (**Table 1** and **Figure 1**). The results clearly suggest that oregano oil can overcome the obstacles of biofilms and kill bacteria within as sufficiently as planktonic bacteria, in contrast to antibiotics that kills bacterial biofilms poorly.

**Table 2:** MIC (mg/ml) of the oregano EO and results of antibiotic susceptibility testing for the pathogens.

Species	Strain no.	Oregano	AMK	AMP	ATM	CFZ	FOX	CRO	CXM	CIP	ERY	GEN	IPM	LVX	MEM	NIT	TET
		
		MIC	R/S	R/S	R/S	R/S	R/S	R/S	R/S	R/S	R/S	R/S	R/S	R/S	R/S	R/S	R/S
*A. baumannii*	AF0004	0.16	R	R	R	R	R	R	R	R		R	R	R	R	R	R
	IQ0012	0.08	R	R	R	R	R	R	R	R		R	S	R	R	R	R
	AF0005	0.16	R	R	R	R	R	R	R	R		R	R	R	R	R	R
	IQ0013	0.16	R	R	R	R	R	R	R	R		R	S	R	R	R	R
*P. aeruginosa*	AF0001	0.64	R	R	R	R	R	R	R	R		R	R	R	R	R	R
	IQ0042	0.56	R	R	R	R	R	R	R	R		R	S	R	R	R	R
	IQ0046	0.64	R	R	R	R	R	R	R	R		R	R	R	R	R	R
	PA01	0.56	R	R	R	R	R	R	R	R		R	R	R	R	R	R
Methicillin-resistant *S. aureus*	AF0003	0.16	R	R		R	R	R			R	S		S		S	S
	IQ0064	0.32	R	R		R	R	R			R	S		S		S	S
	IQ0103	0.32	R	R		R	R	R			R	S		S		S	S
	IQ0211	0.16	R	R		R	R	R			R	S		S		S	S
	USA300	0.32	R	R		R	R	R			R	S		S		S	S


*AMK, amikacin; AMP, ampicillin; ATM, aztreonam; CFZ, cefazolin; FOX, cefoxitin; CRO, ceftriaxone; CXM, cefuroxime; CIP, ciprofloxacin; ERY, erythromycin; GEN, gentamicin; IPM, imipenem; LVX, levofloxacin; MEM, meropenem; NIT, nitrofurantoin; TET, tetracycline; R, resistant; S, susceptible.*

### TEM and SEM Illustrated Ultrastructural Damages of Bacteria

**FIGURE 2:**
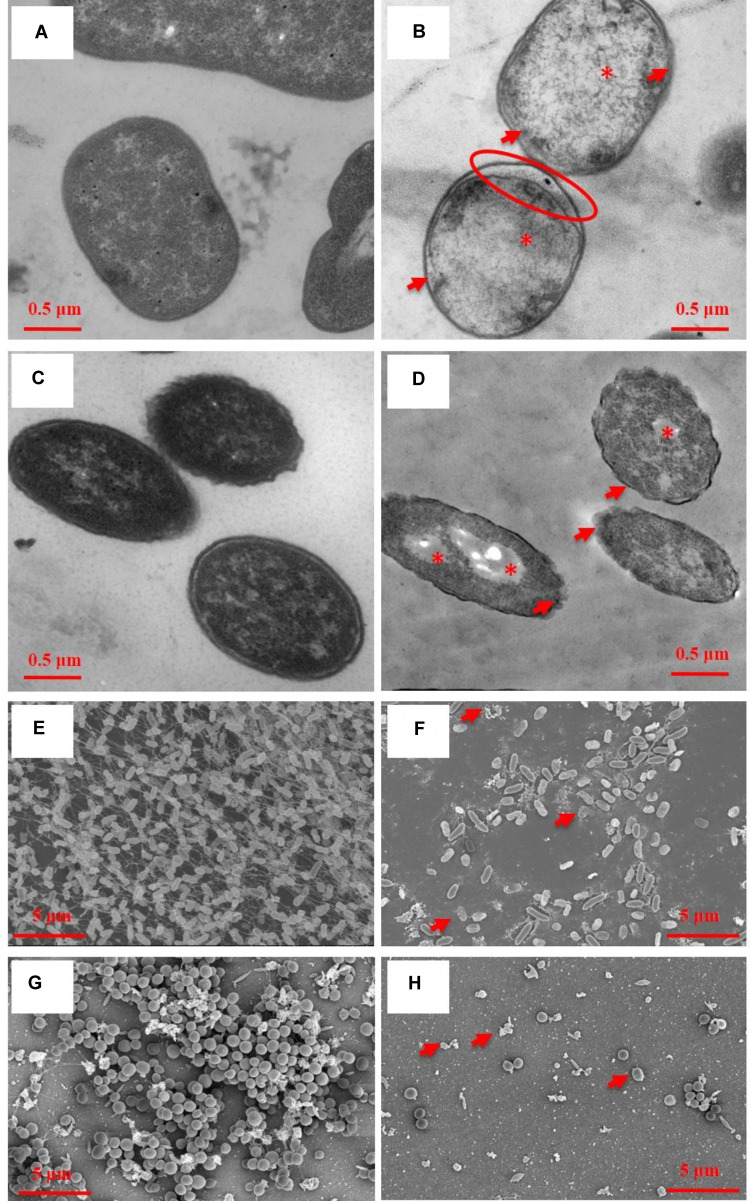
Representative TEM images of planktonic cells **(A–D)** and SEM images of bacterial biofilms **(E–H)** with **(B,D,E,F)** or without **(A,C,E,G)** oregano oil treatment for 1 h. Oregano oil was used at 0.16 mg/ml for *A. baumannii* AF0005 cells, 0.56 mg/ml for *P. aeruginosa* IQ0042 cells, 1.0 mg/ml for *P. aeruginosa* IQ0042 biofilms, and 0.4 mg/ml for MRSA IQ0064 biofilm. **(A,B)** *A. baumannii* AF0005 cells; **(C,D)** *P. aeruginosa* IQ0042 cells; **(E,F)** *P. aeruginosa* IQ0042 biofilms; and **(G,H)** MRSA IQ0064 biofilms. Shown are cell wall and membrane damages **(B,D; arrows)**; dissociation between cell wall and membrane **(B; oval)**, cytoplasmic vacuoles and bubbles **(B,D; asterisk)**, and cell collapse **(F,H, arrows)**. The number of bacteria was drastically reduced after oregano oil treatment in **(F,H)** as compared to untreated controls **(E,G)**.

Transmission electron microscopy showed ultrastructural damages of *A. baumannii* AF0005 (**Figure 2B**) and *P. aeruginosa* IQ0042 cells (**Figure 2D**) after exposure to oregano oil for 1 h at 0.16 mg/ml and 0.56 mg/ml, respectively. The cell wall and membrane damages were apparent in *A. baumannii* AF0005 and *P. aeruginosa* IQ0042 cells with a severe leakage of intracellular substances resulting in cell membrane shrinking and separating from cell wall (**Figures 2B,D**, arrows). Moreover, cytoplasmic vacuoles in *A. baumannii* AF0005 (**Figure 2B**, asterisk) and many stainless-vesicles in *P. aeruginosa* IQ0042 (**Figure 2D**, asterisk) were observed. Intracellular structural discontinuation such as dissociation between cell wall and membrane was also seen in *A. baumannii* AF0005 (**Figure 2B**, oval). In comparison, untreated *A. baumannii* AF0005 (**Figure 2A**) and *P. aeruginosa* IQ0042 cells (**Figure 2C**) had intact, clear cell wall and membrane and dense and homogeneous cytoplasm. However, we did not find any significant differences in the ultrastructure between the control and oregano oil-treated MRSA USA300 by TEM (data not shown), which probably hints at different responses of MRSA from *A. baumannii* or *P. aeruginosa*. As with biofilms, dense and thick bacterial biofilm was observed on the dentin surface, comprised of numerous layers of densely concentrated cocci in 24-h-old *P. aeruginosa* IQ0042 (**Figure 2E**) and MRSA IQ0064 (**Figure 2G**) biofilms. The biofilms of *P. aeruginosa* IQ0042 (**Figure 2E**) and MRSA IQ0064 (**Figure 2G**) were treated with oregano oil at 1 mg/ml and 0.4 mg/ml for 1 h, respectively. The cells decohered in the extracellular polymeric matrix, dead bacteria were readily seen all over, and biofilms were destroyed completely in oregano oil-treated samples (**Figures 2F,H**, arrows). There were only a few bacteria scantly growing on the dentin surface owing to intensive cell death (**Figures 2F,H**).

### No Evidence of Resistant Development to Oregano Oil

**FIGURE 3:**
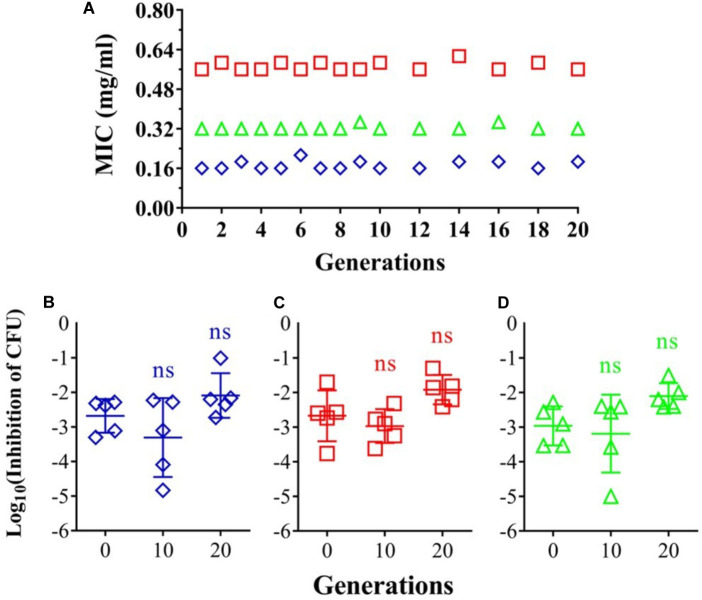
Possible development of bacterial resistance to oregano oil. **(A)** *A. baumannii* AF0005, *P. aeruginosa* IQ0042, and MRSA IQ0064 were passaged for 20 generations each in the presence of indicated MIC doses of oregano oil. The inhibition rate of *A. baumannii* AF0005 **(B)**, *P. aeruginosa* IQ0042 **(C)**, or MRSA IQ0064 **(D)** was unaltered when the bacteria of indicated generations were exposed to oregano oil at an initial MIC concentration. ns, no significance.

Risk of resistant development was evaluated with three representative strains: *A. baumannii* AF0005, *P. aeruginosa* IQ0042, and MRSA IQ0064. The bacteria were cultured up to 20 successive passages in the presence of sub-lethal doses of oregano oil for bacterial inactivation. As shown in **Figure 3A**, *A. baumannii* AF0005, *P. aeruginosa* IQ0042, and MRSA IQ0064 retained susceptibility to the original MIC values of 0.16, 0.32, or 0.56 mg/ml, respectively, after 20 cycles of treatment. Moreover, there were no statistically significant differences in the survival rates of all the bacterial strains among the cycles 0, 1, and 20 in the three tested strains, after exposure to oregano oil at the MICs for 24 h (**Figures 3B–D**). The results indicate that resistance to oregano oil of the bacterial strains did not take place under this condition.

### Oregano Oil Significantly Reduced Bacterial Burden in Burn Wounds Infected With PA01

**FIGURE 4:**
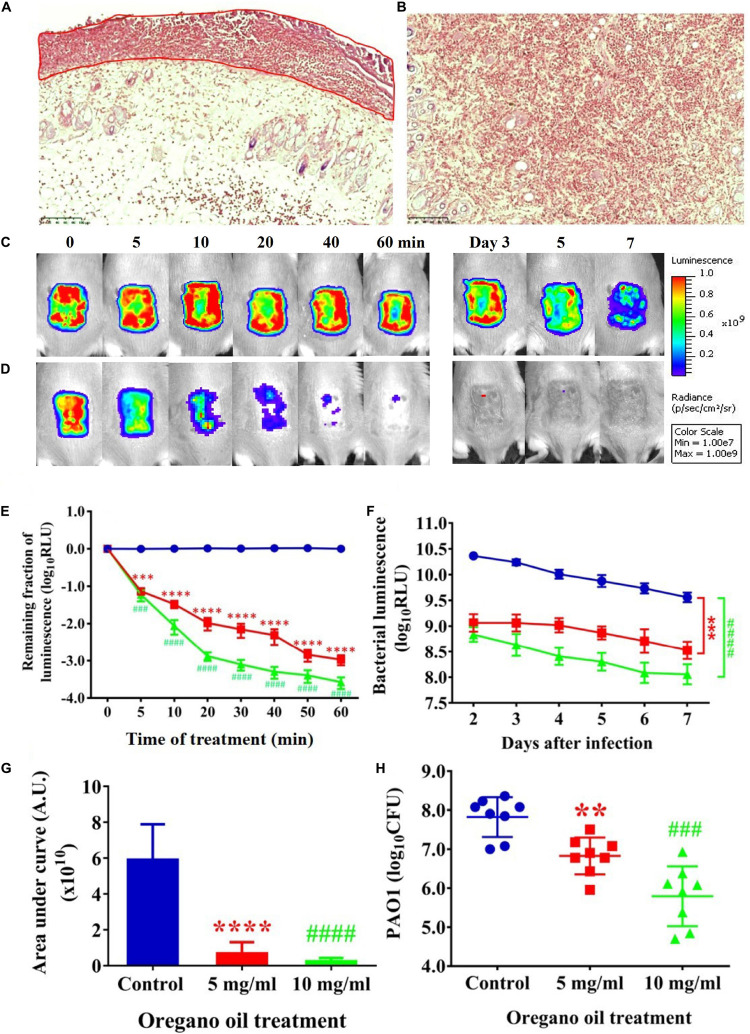
Oregano oil treatment of PA01 infections in the burn wounds. **(A,B)** Gram-stained longitudinal section **(A)** and crossing section **(B)** of a representative wound showing the presence of PA01 biofilms outlined in red. The skin sample was harvested 24 h after bacterial inoculation. **(C,D)** Successive bacterial luminescence images of representative wounds infected with 5 × 10^6^ CFU of luminescent PA01 with **(D)** and without **(C)** oregano oil at 10 mg/ml. The oregano oil was topically applied onto the wounds at 24 h after bacterial inoculation. **(E)** A dose response of mean bacterial luminescence of the wounds infected with 5 × 10^6^ CFU of PA01 in the presence or absence of oregano oil treatment at 5 or 10 mg/ml. **(F)** Time courses of mean bacterial luminescence of the infected wounds in the presence or absence of oregano oil treatment at 5 or 10 mg/ml from days 2 to 7. **(G)** Mean areas under the bacterial luminescence curves **(F)**, representing the overall bacterial burden of infected wounds. **(H)**. The wounds were treated with grape seed oil (control) or oregano oil 24 h after infection and bacterial CFU were quantified on day 7 after bacterial inoculation. RLU, relative luminescence units; A.U., arbitrary units. The data represent means ± SDs (*n* = 8). ^∗∗^*p* < 0.01, ^###^ or ^∗∗∗^*p* < 0.001 and ^####^ or ^∗∗∗∗^*p* < 0.0001 in the presence vs. absence of oregano oil. ns, no significance.

Gram stain of histological longitudinal section (**Figure 4A**) and crossing section (**Figure 4B**) of a representative skin specimen demonstrated the presence of PA01 biofilms at 24 h after bacterial inoculation, as evidenced by abundant bacteria densely clustered together (red) in a highly hydrated extracellular matrix on the surface of skin and in the epidermis (**Figure 4A**, outline in red). In addition, highly resilient microbial assemblies were readily found in the dermis suggesting that the PA01 could infect not only the epidermis but also the dermis within 24 h (**Figure 4B**). In successive bacterial luminescence images of representative wounds infected with 5 × 10^6^ CFU of PA01, oregano oil treatment at 10 mg/ml almost completely eradicated bacterial luminescence in 60 min, while luminescence remained unchanged during the same period in untreated mice (**Figures 4C,D**). Moreover, there was no recurrence of infection in the following days in oregano oil-treated mice, whereas the mice remained significantly infected in untreated mice in the same experimental period (**Figures 4C,D**). An average reduction in bacterial luminescence of 2.9 log_10_ and 3.5 log_10_ were achieved in 60 min at a concentration of 5 or 10 mg/ml of oregano oil, respectively (**Figure 4E**). On the contrary, bacterial luminescence of the wounds in the absence of oregano oil treatment was almost unaltered with only 0.08 log_10_ reduction during the equivalent period (**Figure 4E**, *P* < 0.0001). A time course study of the mean bacterial luminescence from days 2 to 7 after bacterial inoculation corroborated that the treatment consistently and significantly lowered the luminescence compared to untreated mice during the whole period of the experiment regardless of whether oregano oil was used at 5 or 10 mg/ml (**Figure 4F**). The mean areas under the curve (AUC) of the bioluminescence time course were 6.9 × 10^9^ and 2.4 × 10^9^ for oregano oil-treated groups at 5 and 10 mg/ml, respectively, but it was 5.9 × 10^10^ for untreated mice (*P* < 0.0001; **Figure 4G**), representing an 8.6-fold or a 24.6-fold reduction of the AUC in infected burns by oregano oil. We next excised the infected wounds on day 7 to determine bacterial CFU remaining in the wounds. There were 6.7 × 10^6^ and 2.4 × 10^6^ CFU in the wounds treated with oregano oil at a concentration of 5 or 10 mg/ml, respectively, which was significantly lower than the bacterial burden of 6.6 × 10^7^ CFU/wound in untreated mice (**Figure 4H**).

### Oregano Oil Significantly Reduced Bacterial Burden in Burn Wounds Infected With USA300

**FIGURE 5:**
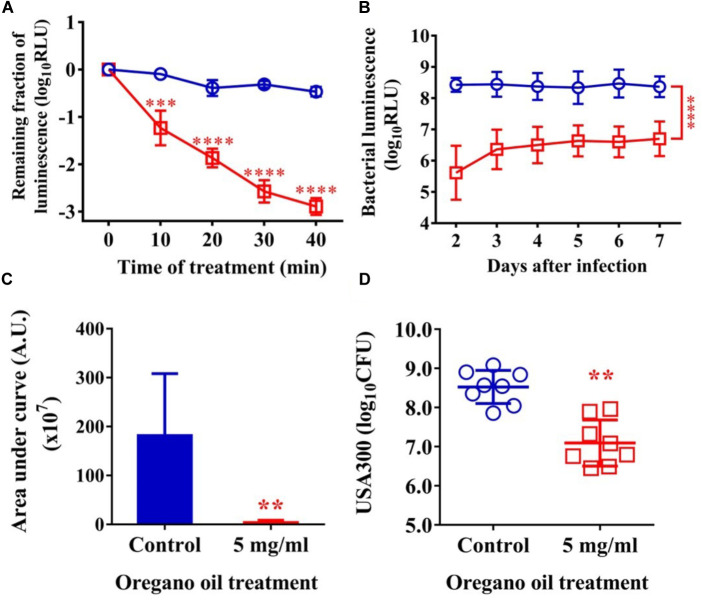
Oregano oil treatment of MRSA USA300 in infected wounds. **(A)** The change in mean bacterial luminescence of the wounds infected with 5 × 10^6^ CFU of USA300 with or without 5 mg/ml oregano oil treatment. **(B)** Time courses of mean bacterial luminescence of the infected wounds with and without oregano oil treatment. **(C)** Mean areas under the bacterial luminescence curves in **(B)**. **(D)** The wounds were treated with grape seed oil (control) or oregano oil once 24 h after infection and bacterial CFU were quantified on day 7 after bacterial inoculation. The data represent means ± SDs (*n* = 8). ^∗∗^*p* < 0.01, ^∗∗∗^*p* < 0.001, and ^∗∗∗∗^*p* < 0.0001 in the presence vs. absence of 5 mg/ml of oregano oil.

As shown in **Figure 5A**, an average reduction in bacterial luminescence of 2.9 log_10_ was attained when the infected wounds were treated with oregano oil at 5 mg/ml for 40 min, which was highly significant compared to only 0.4 log_10_ decline of the bioluminescence in the absence of oregano oil during the same period (*P* < 0.0001; **Figure 5A**). The diminished bacterial luminescence was persistent for 7 days after a single oregano oil treatment at 5 mg/ml (**Figure 5B**). The mean AUC of the bioluminescence were 3.7 × 10^7^ in oregano oil-treated group, but it was 1.8 × 10^9^ for untreated mice (*P* < 0.0001; **Figure 5C**), a 48.6-fold reduction of the AUC by oregano oil treatment. In accordance with this, a single dose of oregano oil treatment diminished bacterial load to 2.7 × 10^7^ CFU/mouse that was 18-fold lower than 4.8 × 10^8^ CFU/mouse in the untreated group (*P* < 0.0001; **Figure 5D**).

### No Side Effects in Mouse Skin Caused by Oregano Oil

**FIGURE 6:**
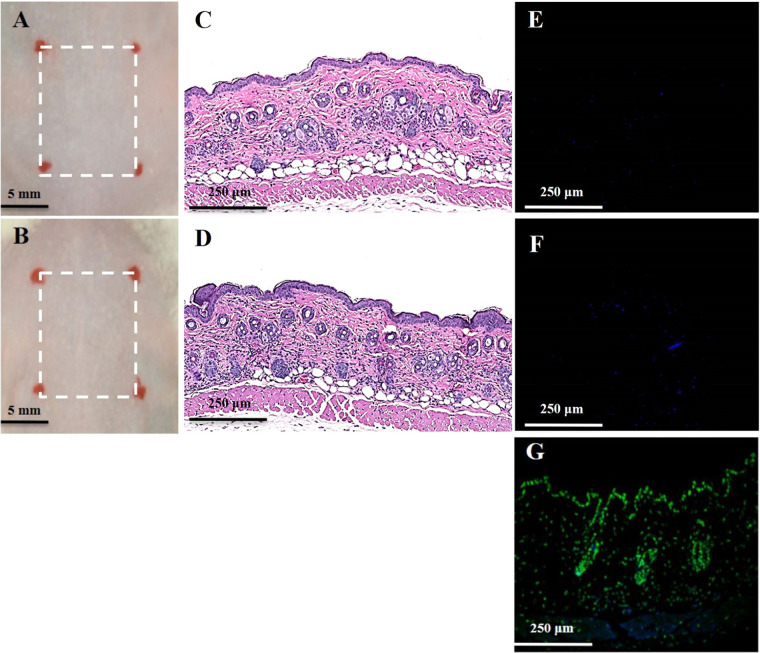
Toxicity evaluation of oregano oil to normal mouse skins. The dorsal skin of mice was topically treated with **(A,C,E)** or without 10 mg/ml oregano oil **(B,D,F)** once a day for three consecutive days. On day 4, the skins were photographed **(A,B)**, followed by histological examination **(C,D)**. The skin sections were also TUNEL stained **(E,F)**. DNase I treated skin samples **(G)** were TUNEL stained in parallel as positive-staining controls.

There was no noticeable skin reaction, as visualized with the naked eye, after three consecutive days of oregano oil treatment at 10 mg/ml (**Figure 6B**) when compared to untreated skin (**Figure 6A**). On the histological levels, the skin maintained an undisturbed structure with a clear layer of healthy epidermal cells on the top of the dermis, indistinguishable to mock-treated skins (**Figures 6C,D**). Genotoxicity was next evaluated by a TUNEL assay to examine any DNA damage induced by oregano oil. In comparison with mock-treated controls, there was no any apparent increase of DNA staining in oregano oil-treated skin (**Figures 6E,F**), while the staining was readily seen in the positive control in which skin section was treated with DNase I (**Figure 6G**). The absence of any oregano oil-induced skin reaction and DNA damage after a 3-day treatment suggests that topical application of oregano oil is neither cytotoxic nor genotoxic to the host.

## Discussion

In seeking non-antibiotic microbicides, we have screened dozens of EOs from Chinese indigenous aromatic plants/spices because EOs have been long recognized as one of the most promising natural products for safe microbicides in folk medicines (Lu et al., 2013a,b,c). The selection was initially based on their antiseptic applications in the food industry and in agricultures after an extensive database search. Among the dozen EOs tested, about one third showed significant antibacterial activities against clinically and agricultural important microbes (Lu et al., 2013a,b,c). Oregano oil stood out as one of the best ones in terms of safety and efficacy. We thus detailed the bactericidal activity of oregano oil against 11 MDR clinical isolates of *P. aeruginosa, A. baumannii* and MRSA as well as two bioluminescent strains of *P. aeruginosa* PA01 and MRSA USA300 in the current study. Oregano oil effectively killed all the bacterial strains tested, with the MICs ranging from 0.08 to 0.64 mg/ml and at an order of sensitivity of *A. baumannii >* MRSA *> P. aeruginosa* (**Table 1**). The finding is in agreement with previous studies demonstrating that oregano oil and its main component carvacrol had a higher MIC against *P. aeruginosa* compared to other species, such as *S.* spp. (Nostro et al., 2007), *Chromobacterium violaceum*, *Salmonella typhimurium*, and *S. aureus* (Burt et al., 2014). Similar to the clinical isolates, oregano oil also inactivated standard strains of *A. baumannii* ATCC 19606 (Rosato et al., 2010), *P. aeruginosa* ATCC 27853 and *S. aureus* ATCC 29213 (Bouhdid et al., 2009), with the MICs 0.15 mg/mL, 1 mg/mL, and 0.33 mg/mL, respectively, which are comparable to our investigation. Previous studies suggested that Gram-negative bacteria appeared to be more resistant than Gram-positive bacteria in response to EO (Tepe et al., 2005; Longaray Delamare et al., 2007; Gilles et al., 2010). This relative resistance of Gram-negative over Gram-positive bacteria may be ascribed to their cell wall structure and outer membrane arrangement. The outer membrane of Gram-negative bacteria is rich in lipopolysaccharide molecules, relatively impermeable to lipophilic compounds, thereby presenting a barrier to penetration of EO antimicrobial substances (Gao et al., 2011). It may be also associated with the enzymes in the periplasmic space, which are capable of breaking down the antimicrobial substances upon their entrance of the cells (Nikaido, 1996). However, our studies disagreed with these observations and found Gram-negative *A. baumannii* was more sensitive to oregano oil than Gram-positive MRSA. This different outcome suggests that antibacterial activity of oregano oil may not depend on the type of Gram reaction in contrast to other EOs, a possibility that is supported by the studies of Gao et al. (2011). In their studies, Gram-negative bacteria *Klebsiella pneumoniae* was the most sensitive bacteria whereas the Gram-positive bacteria *Listeria monocytogenes* was the most resistant strain to the *Sphallerocarpus gracilis* seed EO (Gao et al., 2011).

The possibility that the cell wall and membrane were primary targets of oregano oil was supported by TEM imaging of the ultrastructure of the bacteria. We found damages of the cell wall and membranes, occurrent with cytoplasmic vacuoles, stainless-vesicles, and disruption and discontinuation of the intracellular structures in a large number of bacterial cells after oregano oil treatment (**Figures 2B,E**). This finding is consistent with an association of the antibacterial activity of oregano oil/carvacrol with disturbance of membrane embedded proteins and disruption of lipids, RNA synthesis, ATPase activity, and efflux pump previously demonstrated (Simoes et al., 2009; Tapia-Rodriguez et al., 2017). Moreover, oregano oil may cause an imbalance in intracellular osmotic pressure owing to a leakage of cytoplasmic contents following cell wall and membrane damages, and formation of cytoplasmic vacuoles, eventually inducing cell necrosis, although more investigations are required to conclude the mechanism in detail.

Biofilms are sessile organizations of bacterial cells with a strong adherence to surfaces. Biofilm-associated microbial cells are well protected by an extracellular matrix that comprises exopolysaccharides, proteins and DNA and is poorly permeable (Donlan, 2002; Husain et al., 2015). Systemic antibiotics administered to treat bacterial infections frequently fails at least in part due to the poor permeability of biofilms. Interestingly, oregano oil was capable of biofilm-killing at least at an early stage (24-h-old biofilms) as efficiently as planktonic cells. Biofilms of *A. baumannii*, *P. aeruginosa*, and MRSA were eliminated by oregano oil at a concentration of 0.3, 1.0, or 0.4 mg/ml, respectively, similar to the corresponding MICs attained in planktonic cells. The similarity can be extended to the order of sensitivity with *P. aeruginosa* biofilms more resistant than MRSA biofilms than *A. baumannii* biofilms (**Figure 1** and **Table 1**). This may be attributed to the superior permeability and lipid solubility of oregano oil to bacterial cell membrane and wall (Magi et al., 2015; Khan et al., 2017). Likewise, the effectiveness of oregano oil to inactivate *S. aureus*, *S. epidermidis*, and *P. aeruginosa* biofilms was also found at similar MICs as those against planktonic cells (Nostro et al., 2007; dos Santos Rodrigues et al., 2017; Tapia-Rodriguez et al., 2017). SEM observations confirmed the physical damage and considerable morphological alteration in the *P. aeruginosa* IQ0042 (**Figures 2E,F**) and MRSA IQ0064 (**Figures 2G,H**) biofilms following oregano oil treatment. These observations raise an intriguing possibility that EO may have advantages over water soluble antibiotics in treatment of biofilms because bacteria living in the biofilms are well known to be more resistant to antibiotics (up to 1,000 times) than their planktonic counterparts, in part owing to poor permeability of biofilms to the antibiotics (Ceri et al., 1999; Caraher et al., 2007).

One concern of using oregano oil as an alternative for the treatment of infections in clinics will be whether MDR bacteria can develop resistance to oregano oil. Although this remains largely unaddressed to date, our results suggest that resistance may not be readily developed because 20 passages in the presence of sublethal concentrations of oregano oil did not alter their susceptibility to the oil (**Figure 3A**). Moreover, oregano oil has been used in food prevention and other antiseptic application for centuries and no resistance has been reported so far. It is commonly believed that EOs act at multiple sites within bacterial cells (cell membrane, cell wall, structural proteins, enzymes, nucleic acids, unsaturated lipids, etc.) and would be less likely to induce the development of resistance (Burt, 2004; Simoes et al., 2009; Tapia-Rodriguez et al., 2017). On the contrary, the MIC of conventional antibiotics could gradually increase with a treatment length due to their single action to inactivate the bacteria (Baym et al., 2016; Levin-Reisman et al., 2017).

The bactericidal activity of oregano oil was corroborated in mouse burn models using model bioluminescent strains of Gram-negative *P. aeruginosa* PA01 and Gram-positive MRSA USA300. When applied at 24 h after bacterial inoculation forming early stage biofilms, oregano oil effectively reduced the bacterial burden by 25-folds for PA01 and 49-folds for USA300, respectively, in comparison to untreated wounds. While efficiently inactivating bacteria, oregano oil exhibited no cytotoxicity or genotoxicity to the skin, in good agreement with its long record of safety. Moreover, oregano oil did not adversely affect human keratinocytes (Babili et al., 2011) and was safe when administered orally in mice (Manohar et al., 2001; Preuss et al., 2005; Feng et al., 2017).

In summary, we reported here the effectiveness of oregano oil against a panel of MDR bacteria isolated from combating casualties and demonstrated for the first time efficacy of oregano oil for the treatment of burn infections in mice. The study serves as an initial effort in the pursuit of a novel therapeutic option for wound infections, especially those caused by MDR bacteria.

## Author Contributions

ML designed and performed all the experiments, analyzed the data, and wrote the manuscript. TD supervised and designed the experiments, analyzed the data, and wrote the paper. CM isolated and characterized all the clinical bacteria and wrote the paper. and MW designed and supervised the study, analyzed the data, and wrote the manuscript.

## Conflict of Interest Statement

The authors declare that the research was conducted in the absence of any commercial or financial relationships that could be construed as a potential conflict of interest.
