# Corrigendum: Bactericidal Property of Oregano Oil Against Multidrug-Resistant Clinical Isolates

**DOI:** 10.3389/fmicb.2021.713573

**Published:** 2021-07-12

**Authors:** Min Lu, Tianhong Dai, Clinton K. Murray, Mei X. Wu

**Affiliations:** ^1^Wellman Center for Photomedicine, Massachusetts General Hospital, Harvard Medical School, Boston, MA, United States; ^2^First Area Medical Laboratory, JBSA-Fort Sam Houston, Houston, TX, United States

**Keywords:** oregano oil, *Pseudomonas aeruginosa*, *Acinetobacter baumannii*, MRSA, biofilms, burn wound, mouse model, bioluminescence imaging

In the original article, there was a mistake in Figure 4, panels C and D as published. The images from days 5 and 7 in Figures 4C and 4D are too similar and are not from two days apart (day 5 and day 7). The corrected Figure 4 appears below.

**Figure 4:**
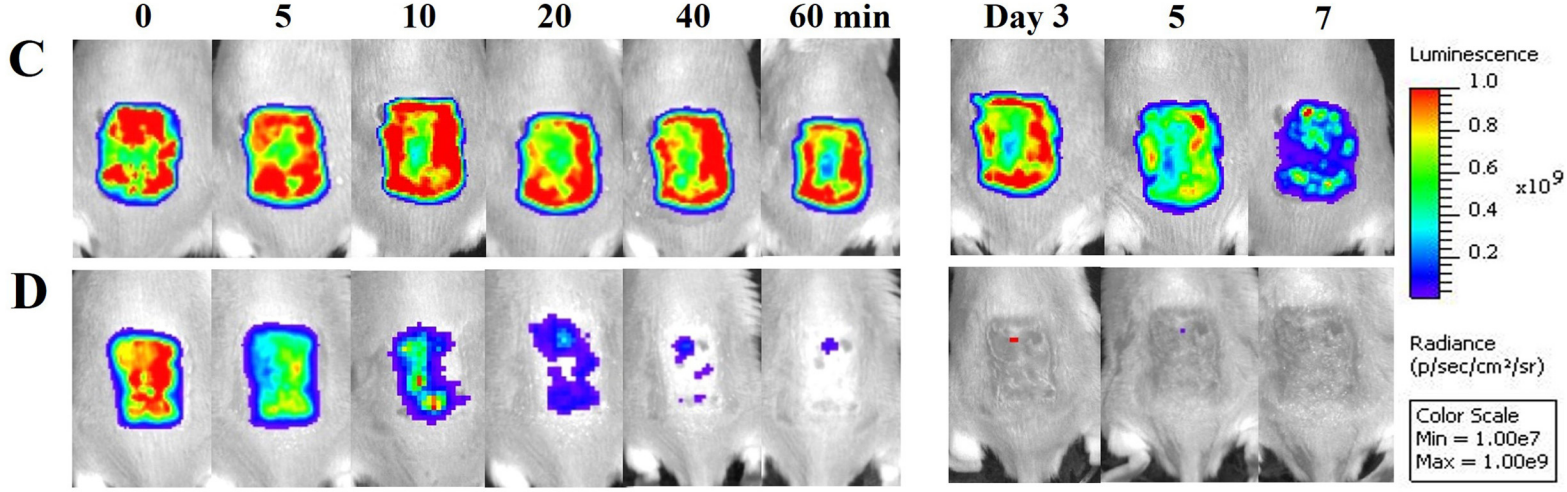
Oregano oil treatment of PA01 infections in the burn wounds. **(A,B)** Gram-stained longitudinal section **(A)** and crossing section **(B)** of a representative wound showing the presence of PA01 biofilms outlined in red. The skin sample was harvested 24 h after bacterial inoculation. **(C,D)** Successive bacterial luminescence images of representative wounds infected with 5 × 10^6^ CFU of luminescent PA01 with **(D)** and without **(C)** oregano oil at 10 mg/ml. The oregano oil was topically applied onto the wounds at 24 h after bacterial inoculation. **(E)** A dose response of mean bacterial luminescence of the wounds infected with 5 × 10^6^ CFU of PA01 in the presence or absence of oregano oil treatment at 5 or 10 mg/ml. **(F)** Time courses of mean bacterial luminescence of the infected wounds in the presence or absence of oregano oil treatment at 5 or 10 mg/ml from days 2 to 7. **(G)** Mean areas under the bacterial luminescence curves **(F)**, representing the overall bacterial burden of infected wounds. **(H)**. The wounds were treated with grape seed oil (control) or oregano oil 24 h after infection and bacterial CFU were quantified on day 7 after bacterial inoculation. RLU, relative luminescence units; A.U., arbitrary units. The data represent means ± SDs (*n* = 8). **p < 0.01, ^*###*^ or ***p < 0.001 and ^*####*^ or ****p < 0.0001 in the presence vs. absence of oregano oil. ns, no significance.

In the original article, there was a mistake in Figure 6, panels C and D as published. Figure 6C was mistakenly duplicated from Figure 6D. The matched images of Figures 6E and F from the same level of tissue slices as Figures 6C and 6D are updated accordingly. The corrected Figure 6 appears below.

**Figure 6:**
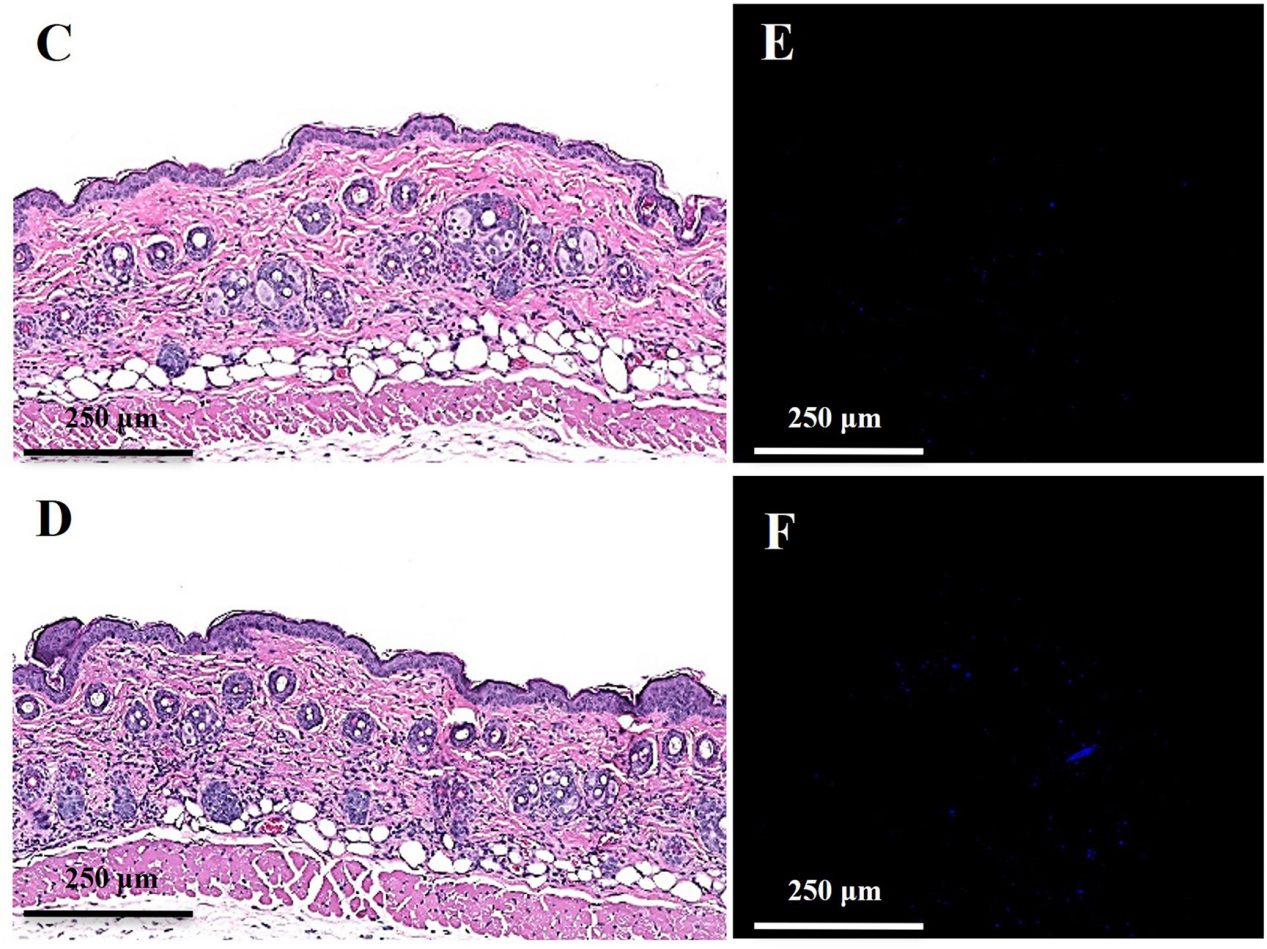
Toxicity evaluation of oregano oil to normal mouse skins. The dorsal skin of mice was topically treated with **(A,C,E)** or without 10 mg/ml oregano oil **(B,D,F)** once a day for three consecutive days. On day 4, the skins were photographed **(A,B)**, followed by histological examination **(C,D)**. The skin sections were also TUNEL stained **(E,F)**. DNase I treated skin samples **(G)** were TUNEL stained in parallel as positive-staining controls.

The authors apologize for this error and state that this does not change the scientific conclusions of the article in any way. The original article has been updated.

